# Mechanism of Enterprise Green Innovation Behavior Considering Coevolution Theory

**DOI:** 10.3390/ijerph191610453

**Published:** 2022-08-22

**Authors:** Xingwei Li, Jiachi Dai, Jinrong He, Jingru Li, Yicheng Huang, Xiang Liu, Qiong Shen

**Affiliations:** 1 College of Architecture and Urban-Rural Planning, Sichuan Agricultural University, Chengdu 611830, China; 2 School of Management, Jiangsu University, Zhenjiang 212013, China

**Keywords:** enterprise green innovation, coevolution theory, green development behavior, regional heterogeneity, meta-analysis

## Abstract

Enterprise green innovation behavior is necessary for the transformation of enterprises and the enhancement of green development. However, the inconsistency of existing studies on the behavioral mechanism has not been effectively addressed. The purpose of this paper is to reveal a mechanism for enterprise green innovation behavior, taking the coevolutionary theory. Based on the coevolution theory model, this study screened 16 high-quality studies covering 11 countries and regions with 5471 independent samples from six major databases (Web of Science Core Collection (SCIE & SSCI), Science Direct, Springer Link, Wiley, Taylor & Francis, and Sage journals). The included literature was coded and tested. Meta-analysis was used to clarify the direction and intensity of the behavioral antecedent and outcome variables to explore the mechanism of enterprise green innovation behavior. Furthermore, this study also explores the moderating effect of regional heterogeneity on behavior. The results are as follows: (1) The economic, political, social, and technological environments significantly and positively influence enterprise green innovation behavior. (2) Enterprises’ green innovation behavior significantly and positively influences environmental performance. (3) Regional heterogeneity can moderate the effects of enterprise green innovation behavior and antecedent and consequence variables. Then, this study proposes countermeasures based on government and enterprise perspectives. This study provides both theoretical and empirical referents for enterprises to better adopt green innovation behaviors and enhance their green development.

## 1. Introduction

As mentioned in the Stockholm+50 International Meeting [1], the three major global environmental crises of environmental pollution, climate change, and biodiversity destruction will worsen at an accelerated rate if the current development model is maintained. Enterprises, as an important party responsible for environmental issues, should part in reversing the eco-situation and development model [2]. According to the theory of the firm [3], a firm is a network of contractual aggregates of stakeholders. Enterprises need to constantly adjust and change their strategies to maximize their own and stakeholders’ interests. Specifically, enterprise green behavior is also a common choice of stakeholders, such as enterprises, governments, and markets, in the face of deteriorating environmental problems. Enterprises adopt green behavior in response to external pressures and internal demand [4]. This leads to the improvement of organizational green performance [5]. As a type of green behavior, enterprise green innovation behavior is considered to be a means of environmental governance by using innovation. Green innovations are technologies, products, and services developed or improved to reduce environmental problems [6,7,8]. Compared with traditional innovation behavior, green innovation behavior includes consideration of environmental issues while achieving economic benefits, such as green product innovation and green technology innovation. Furthermore, the adoption of green innovation behavior by enterprises can not only enhance organizational, economic performance but also positively affect organizational environmental performance [5]. In this process, the government plays the role of supervision and guidance to encourage numerous enterprises to adopt green innovation behavior and improve the standard of green innovation. Specifically, for example, the Chinese government has proposed guidelines on a market-focused green technology innovation system to increase the dominant role of firms [9]. The European Union adopted the proposal of eco-design regulations for sustainable products in 2022, expecting to achieve industrial innovation [10]. Furthermore, enterprise green innovation behavior is also a reflection of market choice. For example, the installed renewable energy market in China exceeded 1 billion kW, accounting for one-third of the global market [11]. Obviously, the huge green market is inseparable from green innovation. Thus, under the new development model, enterprise green innovation behavior has become an important topic of attention for society.

Enterprise green innovation behavior is an effort made by enterprises in the process of adapting to the environment. This behavior is a manifestation of enterprise green development behavior [12,13,14,15,16]. Existing studies have extensively discussed the topic of enterprise green innovation behavior, focusing on the following aspects. The first is studies on the drivers (antecedents) of enterprise green innovation behavior. The group of studies focused on the influence of organizational-level factors (e.g., enterprise managers [17], environmental regulations at the external environment level [18], etc.) on enterprise green innovation behavior. The second is the consequence variables of enterprise green innovation behavior. A group of studies focused on the influence of enterprises’ adoption of green innovation behavior on enterprise value [19], enterprise performance [20], etc. Third, studies consider enterprise green innovation behavior as a mediating variable. For example, Wang et al. (2019) [21] found that green innovation fully mediates the inter-organizational green culture and green performance effect by constructing a structural equation model. Thus, existing studies have made great progress in enterprise green innovation behavior, which has important academic value. However, there are still studies on the factors influencing enterprise green innovation behavior, mostly from a single perspective, and there is no consensus on the research on the paths of enterprise green innovation behavior [22,23,24]. Therefore, further integration analysis has yet to be performed.

Therefore, considering the realistic research context and theoretical research development, this paper presents the core research question: What is the mechanism of enterprise green innovation behavior? The purpose of the study is to reveal the mechanism of enterprise green innovation behavior considering the coevolution theory. Given this, this study selects 16 empirical studies for meta-analysis based on the coevolution theory perspective with the relevant research themes of enterprise green innovation behavior in the period 2007–2021. By doing so, the mechanism of enterprise green innovation behavior around the environment and the organization is investigated to answer the abovementioned research shortcomings. The innovation of this paper is as follows: It expands the research on enterprise green innovation behavior in terms of organization-environment synergy. This provides more theoretical explanations for the path of enterprise green innovation behavior. This study provides both theoretical and empirical referents for enterprises to better adopt green innovation behaviors and enhance their green development. Furthermore, this study applies a new thinking perspective to enhance green development in society.

The follow-up of this study is as follows: First, the theoretical basis and literature review are based on the construction of the hypothetical model. Next, the process of conducting a meta-analysis approach with data sources is described. Then, the results of the meta-analysis of the main and moderating effects are presented, followed by a discussion of the results and managerial insights. Finally, the findings are summarized, and the limitations of the study as well as future research directions are presented.

## 2. Theories and Hypothesis

### 2.1. Theoretical Basis

Coevolution theory considers coevolution to be a reinforcing process of organizational adaptability and resilience [25]. Essentially, coevolution reflects the relationship between the environment and the organization [26]. Lewin et al. [27] introduced coevolution theory into the study of organizations and constructed the first multilevel coevolution framework combining enterprises, industries, and the environment. Flier et al. [25] found that large and mature organizations usually adopt strategic renewal behaviors to adapt to the national institutional environment. Lundan et al. [28] conducted a study on the coevolution of multinational enterprises and local governments in different geographical environments. Furthermore, the development of coevolution theory has shown a multidisciplinary intersection trend between environmental science [24] and economics [29].

**Figure 1:**
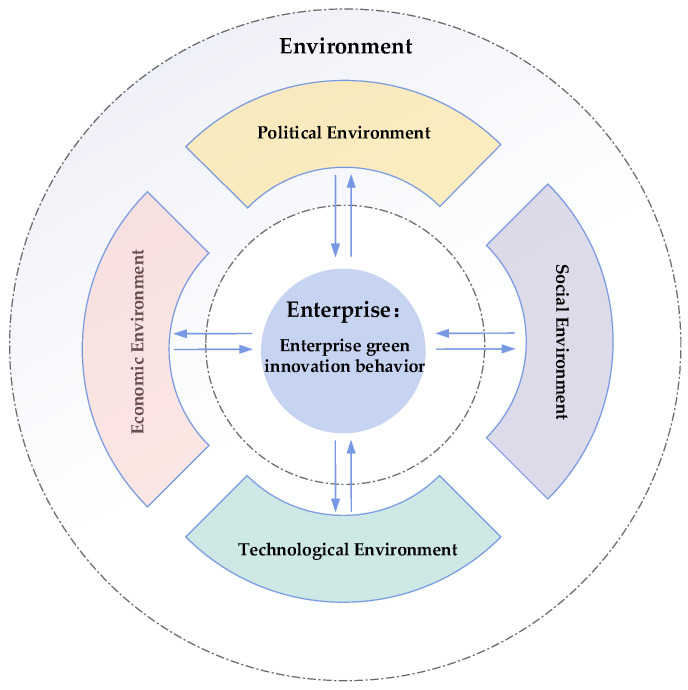
A theoretical model of enterprise green innovation behavior. Note: Summary by authors according to [26,42].

Enterprise green innovation behavior plays a significant positive role in enterprise environmental performance [30], enterprise financial performance [31], and enterprise green image [32]. By combing through the literature, the established research explores the influencing factors of enterprise green innovation behavior mainly in the following aspects: First, market orientation. In the market, green supply chain partners [33], consumers [34], and competing enterprises [35] can influence enterprise green innovation behavior. The second is government orientation. The environmental regulations, subsidies, and tax rate control adopted by the government are all factors that influence the green innovation behavior of enterprises [32,36,37]. Third organizational orientation. Existing studies prove that transformational leadership, social responsibility, the green atmosphere of the organization, and the environmental competence of the enterprise are factors that influence enterprise green innovation behavior [6,38,39,40]. Overall, enterprise green innovation behavior is always influenced by both the environment it is in and the internal aspects of the organization. In other words, enterprises are constantly adapting to both internal and external organizational influences, which is also consistent with coevolution theory. Therefore, this study proposes a theoretical model of enterprise green innovation behavior based on coevolution theory, as shown in Figure 1. The theoretical model reflects the relationship between the internal organization and the environment of the enterprise. Based on PEST (political, economic, social, technological) analysis [41], this paper divides the environment into four dimensions: economic, political, social, and technological.

However, the differences brought by research methods and perspectives have led to a mixed understanding of existing studies on the factors influencing enterprises’ green innovation behavior. Chen et al. [43] stated that environmental regulations set by the government do not promote proactive enterprise adoption of green innovation behavior. However, Lian et al. [36] concluded that environmental regulations positively and significantly influence enterprise green innovation behavior. Singh et al.’s [30] study shows that green transformation leaders indirectly influence enterprise green innovation behavior; therefore, Pham et al.’s [44] findings of a direct significant positive impact of green transformation are inconsistent. This shows that there are differences in the views under single conclusions. Therefore, it is highly necessary to integrate the results of existing studies to reveal the mechanism of enterprise green innovation behavior. This study explores the path of enterprise green development behavior from the perspective of coevolution theory. Furthermore, by combing through the literature, it is found that there are rich research results on the research topics surrounding the mechanism of enterprise green innovation behavior, as shown in Table 1. Observing Table 1, the existing studies [22,23,24,45,46] are not comprehensive in explaining the mechanism of enterprise green innovation behavior. Among them, Tariq et al. [23] integrated the mechanism of enterprise green innovation from a qualitative perspective. The results of this study, although systematic and comprehensive on the mechanism of enterprise green innovation, lack quantitative studies to verify the conclusion. Therefore, there is a strong need to analyze the mechanism from a combined qualitative and quantitative perspective. Qin et al. [22] used meta-analysis to explore the mechanism of enterprise green innovation behavior. However, the study has not yet considered the influence of moderating factors on enterprise green innovation behavior. Li et al. [47] found that regional heterogeneity can moderate the effect of enterprise green development behavior. In addition, regional heterogeneity was shown to moderate the effect of government green development behavior [48]. Arranz et al. [49] used an ordinal logit regression model to confirm that the economy, the distribution, and the number of enterprises in the region where the enterprise is located can promote the eco-innovation of the enterprises. Does regional heterogeneity moderate the effect between enterprise green innovation behavior and antecedent and consequence variables? Therefore, this paper attempts to explore the mechanism of enterprise green innovation behavior systematically and comprehensively from a combined qualitative and quantitative perspective using meta-analysis from the theory of synergistic evolution to provide support for filling the existing research gap.

### 2.2. Hypothesis Development

#### 2.2.1. Environment

PEST analysis is commonly used to analyze potential factors external to the enterprise, and the use of the PEST analysis framework can avoid the omission of potential environmental factors [42,50]. Existing studies have focused on external environmental factors such as consumers, environmental regulations, competing firms, cooperative firms, and sociotechnical levels to influence enterprise green innovation behavior [22,23,24]. Following the PEST analytical framework, the above factors can be classified into four dimensions: economic, political, social, and technological.

The economic environment is mainly represented by two perspectives: the market and the public. The market mainly includes elements involving the economic dimension, such as market mechanism and consumer demand. The public dimension includes elements such as gross domestic product (GDP) and per capita disposable income. Using partial least squares structural equation modeling, Jun et al. (2019) [46] found a significant positive contribution of market and consumer factors to the adoption of green innovation behavior by SMEs (small and medium enterprises) in Pakistan. Li et al. [51] constructed a vector autoregressive model and found that GDP value added drives construction enterprises to implement green innovative technology. Therefore, this study proposes the following hypotheses:

**H1**.  *The economic environment significantly and positively influences enterprise green innovation behavior*.

The core of the political environment lies in the political atmosphere created under the government’s leadership. Li et al. [52] constructed a partial least squares structural equation model and showed that the political and institutional environment significantly and positively influences the green development behavior of industrial enterprises. In this study, the political environment is mainly reflected in environmental supervision and environmental regulation. In terms of government environmental supervision, Wang et al. [53] analyzed panel data and found that the level of environmental supervision by local governments was positively related to the level of green innovation of enterprises. In terms of environmental regulation, Peng et al. [54] constructed a theoretical model and analyzed the effects of different types of environmental regulation on green technology innovation behavior. The results of the study showed that both command-and-control environmental regulation and incentive environmental regulation significantly and positively drive enterprises to adopt green technology innovation behavior. Therefore, this study proposes the following hypotheses:

**H2**.  *The political environment significantly and positively influences enterprise green innovation behavior*.

The social environment mainly includes demographic characteristics. Such as population, demographic structure, and customs. Furthermore, corporate social responsibility (CSR) connects the social environment and the enterprise. Considering social issues in production and operation, building a green and sustainable corporate image is a manifestation of social responsibility. Ji et al. [55] analyzed panel data to reveal the mechanism by which CSR influences collaborative innovation in developing countries. Among them, the CSR of the environment positively influences collaborative innovation. Therefore, this study proposes the following hypotheses:

**H3**.  *The social environment significantly and positively influences enterprise green innovation behavior*.

The technological environment includes technologies, such as patents and professional knowledge. Based on the generalized ordinal logic model, Triguero et al. [56] found that one of the key factors in improving green innovation is the importance of technological capabilities. Furthermore, Roh et al. [57] used partial least squares structural equation modeling and found that intellectual property rights have a significant positive driving effect on green innovation in Korean manufacturers. Therefore, this study proposes the following hypotheses:

**H4**.  *The technological environment significantly and positively influences enterprise green innovation behavior*.

#### 2.2.2. Enterprises

Enterprise performance is a quantification of the business situation of an enterprise. Among them, it relies heavily on the enterprise’s environmental performance in terms of environmental management. Environmental performance is the quantitative result of a enterprise environmental behavior [58,59]. Existing studies on enterprise green innovation behavior have focused on exploring its relationship with the enterprise environmental performance. Singh et al. [30] constructed a partial least squares structural equation model and verified that green innovation behavior could predict enterprise environmental performance. Li et al. [60] pointed out that enterprise green innovation behavior consists of three dimensions: green product innovation, recycling, and green advocacy. Among them, green advocacy positively affects environmental performance. Therefore, this study proposes the following hypotheses:

**H5**.  *Enterprise green innovation behavior significantly and positively affects environmental performance*.

#### 2.2.3. Moderator

Regions tend to exhibit green diversification and different adaptive capacities [61]. In other words, there are also large differences in the development of green innovation activities in different regions. Wang et al. [62] measured the regional green development levels in 30 Chinese provinces and found that there is variability in regional green development levels. Zhang et al. [63] found that the effect of regions with different carbon emission intensities on green technology innovation was inconsistent. The higher the carbon emission intensity is, the stronger the inhibition of green technology innovation. In summary, this study considers that regional heterogeneity can moderate the effects of enterprise green innovation behavior and antecedent and consequence variables. For example, regional heterogeneity may moderate the relationship between the economic environment and enterprise green innovation behavior. Therefore, this study proposes the following hypotheses:

**H6**.  *Regional heterogeneity can moderate the effects of enterprise green innovation behavior and antecedent and consequence variables*.

**Figure 2:**
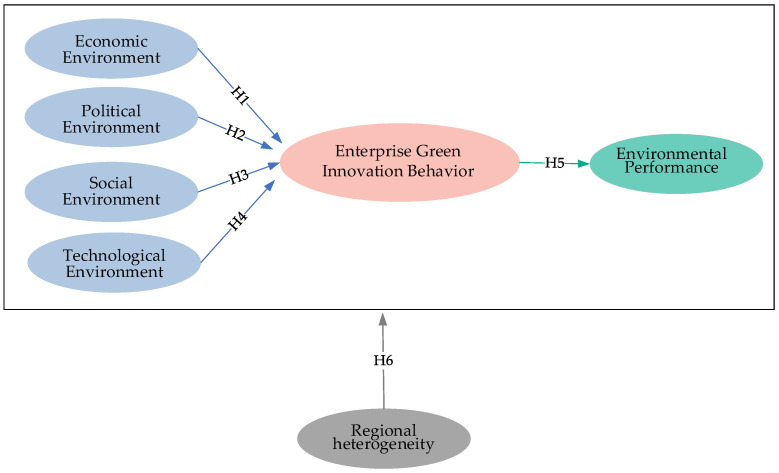
A hypothetical framework for enterprise green innovation behavior.

In general, this paper proposes a hypothesis framework, as shown in Figure 2.

## 3. Method and Data

### 3.1. Meta-Analysis

A meta-analysis, an important statistical analysis tool, performs statistical analysis by synthesizing data from multiple studies [64]. Early in the development of meta-analysis, the method adopted predetermined mathematical standards to statistically analyze research in medicine [65], education [66], and other related disciplines. With the development of interdisciplinarity, it is extensively cited in environmental science [49], management [48], and other disciplines. The main reasons for this paper to reveal the mechanism of enterprise green innovation behavior based on meta-analysis are as follows: First, unlike other traditional statistical analysis methods, meta-analysis is a quantitative analysis method that follows predefined screening criteria. Thus, the results of this study eliminate the bias caused by subjective judgment. Second, existing empirical studies vary in the effect of mechanism, strength, and significance. In summary, this study takes green innovation behavior as the research object and collects relevant empirical studies using objective screening criteria. Furthermore, this study uses meta-analysis, which uses statistical analysis methods to comprehensively analyze the mechanism of enterprise green innovation behavior. It provides some reference value for enterprises to realize green innovation behavior and enhance the green development of society.

#### 3.1.1. Data and Code

Based on the Web of Science Core Collection (SCIE & SSCI), Science Direct, Springer Link, Wiley, Taylor & Francis, and Sage journals, this study was searched to ensure that the data were as reliable and representative as possible. Schiederig et al. [67] showed that green innovation, eco-innovation, environmental innovation, and sustainable innovation could be used interchangeably. Therefore, to screen the related research on green innovation behavior as comprehensively as possible, this paper uses green innovation, eco-innovation, environmental innovation, sustainable innovation, and sustainable innovation as subject headings to conduct a full-text search. Referring to Takalo et al. [68], the time interval of this study was set to 2007–2021. The search for this study was conducted on 15 July 2022, and a total of 2845 relevant papers were collected.

Furthermore, according to the screening criteria established by the mature meta-analysis literature [69,70] and the characteristics of this study, this paper established the following screening criteria: (1) The included studies must be empirical studies. (2) The included studies must include the subject of this study on enterprise green development behavior. (3) The included studies must report the effect size as well as the sample size. (4) The included studies must be independent samples.

**Figure 3:**
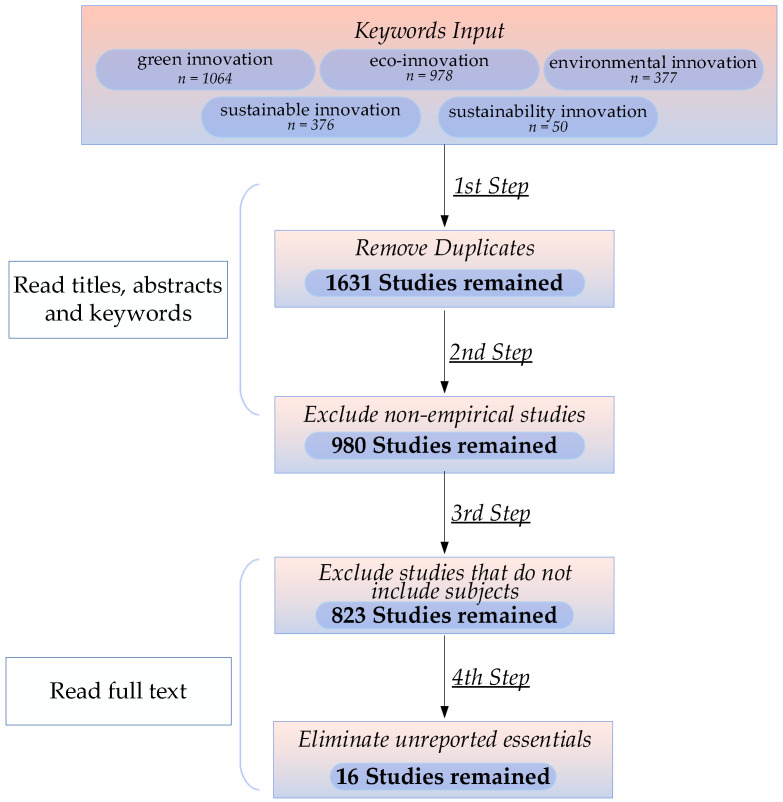
Screening steps for target literature.

Based on four screening criteria, this paper conducts the following screening (as shown in Figure 3). To reduce the influence of errors caused by subjective judgments, this paper strictly follows the mature screening criteria, and two researchers independently carried out the screening steps and compared the screening results. In the 1st step, 1214 duplicate papers were removed by reading the titles, abstracts, and keywords of the collected 2845 papers on green innovation behavior-related studies. After removing duplicates, 1631 papers remained. In the 2nd step, 651 nonempirical papers were removed by reading the titles, abstracts, and keywords of 1631 papers. After removing nonempirical papers, 980 papers remained. In the 3rd step, by reading the full text of 980 papers, 157 papers were excluded from the research subjects that did not include enterprise green innovation behavior. After removing the papers that included the research subject of enterprise green development behavior, 823 papers remained. In the 4th step, by reading the full text of 823 papers to screen out studies that did not report complete effect sizes, sample sizes, and nonindependent samples, 807 papers were excluded. Finally, this study included 16 empirical studies, which met the minimum sample size requirement of 10 for meta-analysis [71].

A commonly used analytic metric for meta-analysis is the effect size, which is used to measure the strength of the effect [72]. By looking at the 16 included samples, it was found that the effect sizes were all measured using Pearson correlation coefficients (*r*). However, the Pearson correlation coefficient, as an equidistant scale, cannot be simply averaged. Therefore, it is often necessary to convert r to Fisher’s *Z* before performing the meta-analysis. The Pearson correlation coefficient after transformation to Fisher’s *Z* satisfies the normal distribution. Based on [73], this study converts Pearson’s correlation coefficient, which cannot be simply averaged, to Fisher’s *Z*. The conversion was done as follows:

(1) Z=12×ln[1+r1−r] 

(2) SEz=1N−3 

where *Z* represents Fisher’s *Z*, *r* represents the Pearson correlation coefficient, *SEz* represents the standard error of Fisher’s *Z*, and *N* represents the sample size. In this study, the coding sheet was divided into two major parts according to the content and purpose of the study. The first part was divided into basic information, including authors, years of publication, and study variables. The second part was the statistical information, including Fisher’s *Z* and sample size. As shown in Table 2, it involved 22 effect sizes and 5471 sample sizes. Among them, the sample size involved in Roh et al. [57] is 1203, which is 21.99% of the total sample.

#### 3.1.2. Publication Bias Test

**Figure 4:**
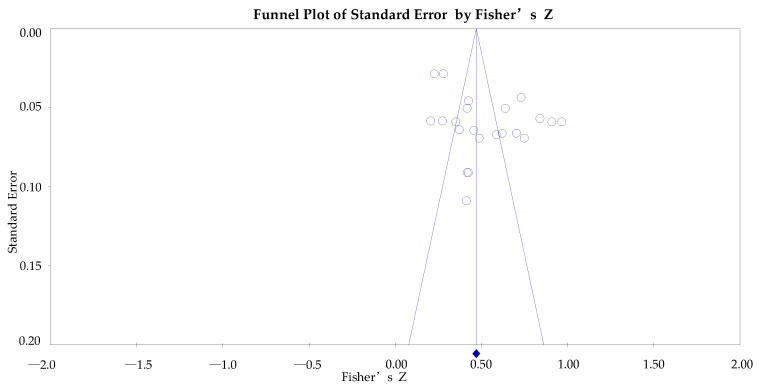
Total sample funnel chart.

Studies that could not be included may introduce publication bias to the results of this study, for example, non-English literature, tentatively unpublished literature, and gray literature. In general, funnel plots are used in meta-analysis methods to test for publication bias. The horizontal axis of the funnel plot is the effect size, and the vertical axis is the standard error. Included studies are distributed as dots on either side of the midline (i.e., the vertical line corresponding to the mean effect size). When the distributions of the included studies were symmetric about the midline as the axis of symmetry, this indicated that there was no bias. In this study, the funnel plot was output by Comprehensive Meta-Analysis v3 (Biostat Inc., Englewood, NJ, USA), as shown in Figure 4. The included studies were not symmetrically distributed on either side of the midline and were skewed. This suggests that this study may be biased. Furthermore, funnel charts are only a visual tool to identify publication biases, which are more subjective intuitively. Therefore, further confirmation of publication bias from a quantitative perspective is needed.

Rosenthal’s fail-safe N, Begg and Mazumdar rank correlation, and Egger’s regression intercept are tests to quantify publication bias. It is assumed that there is no publication bias in this study. Next, the study further examined publication bias, and the results are shown in Table 3. Rosenthal [85] proposed the fail-safe N calculation method to test publication bias and judged whether it was statistically significant by calculating the *p*-value corresponding to the combined effect. Rosenthal’s fail-safe N test shows that the *p*-value < 0.001 has statistical significance. Meanwhile, when α = 0.05, the Z-value > 1.96. This indicates that there is no publication bias in this study. The test results reject the null hypothesis. Begg and Mazumdar rank correlation tests for publication bias by calculating the correlation between variance and effect size [86], a method that does not require any model assumptions. Publication bias is indicated when the test results are statistically significant. The results show that the *p*-value (2-tailed) > 0.05. That is, there is no publication bias in this study. The test results reject the null hypothesis. Egger’s regression tests for publication error by linear regression analysis [87]. When the test results are significant, this indicates that the study has publication bias. This shows that there is no publication bias. The test results reject the null hypothesis. In summary, the results of the above tests indicate that there is no bias in this study.

#### 3.1.3. Heterogeneity Test

The different methodologies and data sources involved in the included literature may have made differences in the results of this paper. Therefore, it is highly essential to conduct a heterogeneity test. It is assumed that the actual effect size of this study is consistent with the expected effect size, i.e., there is no heterogeneity. The Q statistics reflects the difference between the actual and expected effect sizes, and I² reflects the proportion of the total effect size that is heterogeneous [88]. Therefore, this study used the Q statistics and I^2^ to identify and quantify the heterogeneity of the study, and the test results are presented in Table 4. The magnitude of the difference between Q and df (degrees of freedom) reflects the degree of heterogeneity of the effect size. The Q statistics results show that Q (405.242) > df (21) and the *p*-value < 0.050; that is, there is significant heterogeneity in the study, and the original hypothesis is not valid. The I² values are taken as 25%, 50%, and 75% as low, high, and high, respectively. 50% and 75% are divided into low, medium, and high heterogeneity intervals [89]. The closer I^2^ is to 100%, the higher the heterogeneity of the study. The I^2^ was 94.818%, which is in the high heterogeneity interval. This shows that the hypothesis is not valid, and the results of this test indicate that this study is highly heterogeneous. Therefore, the random effects model was used in this study to avoid the bias caused by heterogeneity.

#### 3.1.4. Outlier Test

**Figure 5:**
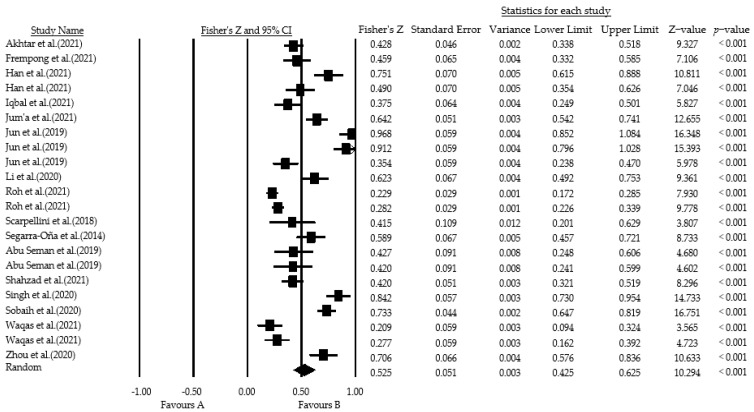
Total sample forest plot [30,45,46,57,60,74,75,76,77,78,79,80,81,82,83,84].

Forest plots can visualize the point estimates and confidence intervals of effect sizes for each study, thus discriminating outlier studies [90]. The area of the black box reflects the weight attributed to this study, and the vertical line indicates the interval of the point estimate. When the vertical line crosses the 0-scale line (i.e., invalid line), it indicates that the effect size is not significant and is an outlier. As shown by the forest plot in Figure 5 of this study, there were no outlier studies included in this study.

#### 3.1.5. Sensitivity Analysis

**Figure 6:**
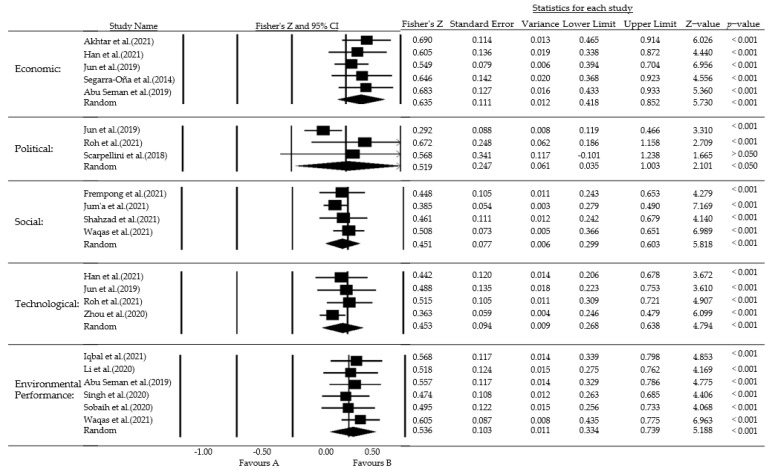
Sensitivity analysis of each dimension [30,45,46,57,60,74,75,76,77,78,79,80,81,82,83,84].

In this paper, the method of excluding one study at a time was used to test the robustness of the results of the meta-analysis. The results of the test are shown in Figure 6. In the economic environment dimension, the effect values before and after exclusion are in the same direction and remain in the range of 0.549–0.690. This indicates that the results of the economic environment dimension are robust. In the political environment dimension, after excluding Scarpellini et al. (2018) [79] (Fisher’s Z = 0.568, *p*-value = 0.096), the findings change to a nonsignificant level, which indicates that the dimension is not robust and that the study should be excluded. In the social environment dimension, the effect values before and after exclusion were in the same direction and remained in the range of 0.385–0.508. This indicates that the results of the social environment dimension are robust. In the technological environment dimension, the effect values before and after exclusion are in the same direction and remain in the range of 0.363–0.515. This indicates that the findings of the technological environment dimension are robust. In the environmental performance dimension, the direction of the effect values before and after exclusion is consistent, remaining in the range of 0.256–0.435. This indicates that the results of the environmental performance dimension are robust.

## 4. Results and Discussion

### 4.1. Analysis and Discussion of Antecedent and Consequent Effects

The main effect results show (see Table 5) that enterprise green innovation behavior is significantly and positively related to the environment (0.521) and the enterprise (0.536). The results of this study support hypothesis H1–H5. Overall, the antecedent and consequent variables of enterprise green development behavior are positive. Among them, the economic environment has the largest effect size with enterprise green innovation behavior. In other words, the economic environment has the strongest degree of effect on enterprise green innovation behavior.

How does the green innovation behavior of enterprises behave in the context of the environment they are in? The economic environment (0.635), political environment (0.519), social environment (0.451), and technological environment (0.453) are all significantly and positively related to enterprise green innovation behavior. In other words, enterprise green innovation behavior is influenced by economic, political, social, and technological environments. This finding also complies with Jun et al. [46] and Roh et al. [57].

Overall, enterprises choose to adopt a strategy of green innovation behavior when they are positively influenced by the external environment. The possible root cause of this situation is that the external environment puts pressure on enterprises on four levels: economic, political, social, and technological. At the economic level, enterprises are always dependent on markets and consumers for their operations. The market system is gradually tilting toward green markets. Consumer demand for green products is increasing. Supply chain partners are expanding green product offerings. Competing enterprises are increasing their green technology research and development efforts. All four of these put tremendous economic pressure on enterprises. Thinking about market share, stable operation, and competitive advantage, enterprises must adopt green innovative behavior. At the political level, laws, regulations, and related systems have improved under the leadership of the government. Due to the contradiction between economic development and environmental protection, the government has given top priority to green development. Enterprises are a key part of the process of achieving green development. The government relies on environmental regulation and supervision to guide enterprises to adopt green development behaviors and increase green innovation using incentives, penalties, and coercion. At the social level, against the background of gradual environmental deterioration and government propaganda, the public is also keeping a close watch on green development. The public is involved in the green development process of enterprises using supervision and reporting. Similarly, to respond to public supervision and reporting, enterprises have to increase their green innovation efforts and focus on establishing their social green image. At the technical level, green innovation behavior cannot be separated from green innovation technology. Universities and R&D institutions are constantly upgrading the industry’s green innovation technologies. In the process of technology updating and iteration, enterprises are prompted to develop and use green innovative technologies.

At the enterprise level, the complexity within the organization makes it less easy for enterprises to adopt green innovation behaviors. As far as organizational decision makers are concerned, the decision makers’ own historical experience, green preferences, profitability, etc., will all affect enterprise green innovation behavior. Therefore, environmental performance is particularly prominent in this context. The two major labels for environmental performance are environmental management and enterprise performance. Both of these are key focus points for decision-makers. Not only that, but also the green climate that decision-makers create within the organization is extremely important. For the organization’s employees, both their characteristics and the organization’s green climate are important factors that trigger the organization’s employees to lean toward green innovation behaviors.

### 4.2. Moderator Analysis and Discussion

Table 6 shows the results from the moderator. The between-group Q-value (260.334) > df (9), *p*-value < 0.001. This suggests that regional heterogeneity, a moderating variable, significantly reduces between-group heterogeneity. In other words, regional heterogeneity moderates the relationship between variables, which indicates that the results of this study support hypothesis H6. This result is in agreement with Santoalha et al. [61]. In particular, Jordan (0.912, *p*-value < 0.001) corresponds to the largest effect size, followed by the United Arab Emirates.

## 5. Conclusions

### 5.1. Main Findings

Based on the coevolution theory, this paper constructed a theoretical model of enterprise green innovation behavior at the environmental and organizational levels. Then, the meta-analysis was used to explore 16 high-quality empirical studies from 2007 to 2021, which provided enlightenment for the theoretical model of enterprise green innovation behavior. In addition, the moderating effect of regional heterogeneity on green innovation behavior is discussed.

The main findings of the research are as follows:

(1) The economic, political, social, and technological environments significantly and positively influence enterprise green innovation behavior. The effects of the economic, political, social, and technological environments are not consistent. Among them, the economic environment has the greatest impact on enterprise green innovation behavior, and the political environment has the second highest impact.
(2) Enterprise green innovation behavior significantly and positively affects environmental performance.
(3) Regional heterogeneity can moderate the effects of enterprise green innovation behavior and antecedent and consequence variables.

### 5.2. Policy Implications

This study revealed the mechanism of enterprise green innovation behavior from the perspective of environmental and organizational coevolution. In this regard, the government is an important participant in the process of an enterprise adopting green innovation behavior. The main manifestations are that the government improves the green market system, strengthens public green awareness, and improves the environmental governance system and environmental laws and regulations. For this reason, this study proposes management inspirations for enterprises’ adoption of green innovation behaviors from the perspectives of government and enterprises.

The governmental perspective is divided into three parts: environmental regulation, environmental supervision, and R&D investment. First, the government needs to develop better environmental regulations around market trends. The government gradually realizes an environmental regulatory system oriented to the improvement and activation of the market. In addition, the government should consider regional heterogeneity and develop environmental regulations for each region’s development. Environmental regulation is considered to be an important tool to drive enterprises to adopt green innovation behaviors. A large number of governments have continued to introduce environmental regulations to improve the environmental governance system. According to the International Energy Agency [91], there are 253 existing environmental regulations in the United States. In some countries, there are fewer than 10 environmental regulations. Furthermore, more appropriate environmental regulations should be developed for different industries [54,92]. For example, manufacturing industries face more severe environmental problems than lighter environmental problems, such as technology and education industries. Manufacturing enterprises, then, may need more favorable environmental taxes to mitigate the cost of their adoption of green innovation behaviors. Second, the government can increase the intensity and frequency of environmental supervision. Furthermore, the realization of a transparent environmental supervision system is also extremely necessary. Through the disclosure of environmental regulatory information, the government enables all sectors of society to participate in the process of environmental regulation to promote the level of enterprise green innovation. The fines for environmental administrative penalties in China exceeded 8.24 billion yuan in 2020 [93]. The level of environmental supervision cannot be improved without the joint participation of the central government and local governments. The central government sends central environmental inspection teams to cooperate with local governments to carry out environmental regulations at the local level to help enterprises achieve green innovation. Third, the government needs to continuously raise the investment in green innovation R&D. On this basis, the government also needs to consider how to effectively monitor the flow and use of R&D funds invested in green innovation enterprises. When the inflow and output of green innovation enterprises are unsatisfactory, the government needs to decide whether to take appropriate action to increase investment efforts or to stop losses in time. The U.S. The Environmental Protection Agency (EPA) Science and Technology Account for Science and Technology Research and Development was allocated $778.1 million in 2021 [94]. In the process of green innovation, enterprises need to involve a large amount of money in green technology development. The government enhances the investment in R&D of green innovation technology, which can not only reduce the pressure of R&D investment of enterprises but also improve the technology level of the industry. Ultimately, it will enhance the level of green technology innovation across society.

The enterprise perspective mainly considers two perspectives: managers and grassroots employees. Managers should change their attitudes. When organizing the production and operation of an enterprise, managers should abandon the primacy of profit and instead give top priority to environmental protection. In addition, managers should focus on creating a green atmosphere within the organization. This will effectively guide grassroots employees to participate more actively in the green innovation process. As far as grassroots employees are concerned, they should not only cultivate their green values but also actively participate in green innovation.

### 5.3. Theoretical Contribution

The theoretical contribution of this study is to enrich the research field of coevolution theory. This study has explored the mechanism of enterprise green innovation behavior by using coevolution theory, which provides a new perspective for understanding coevolution theory. On the other hand, this study uses meta-analysis to integrate the studies related to enterprise green innovation behavior systematically, verifies the mechanism of enterprise green innovation behavior, and extends the theoretical study of enterprise green development behavior.

### 5.4. Research Limitations and Perspectives

Similarly, this study has some limitations. First, this paper explored the mechanism of enterprise green innovation behavior from the environmental and organizational perspectives. However, due to space limitations, this study only considered the dimension of environmental performance at the organizational level. Future studies should have considered expanding the dimensions at the organizational level. Second, due to the limitations of the sample during the collection of the study, this study only established a moderating effect. Future studies may consider other potential moderating variables. These include industry differences, firm size differences, firm competition, and firm cooperation.

## Figures and Tables

**Table 1:** Research Gap.

Researcher	Economic Environment	Political Environment	Social Environment	Technological Environment	Environmental Performance	Moderator	Quantitative Research
Tariq et al. (2017) [23]	√	√	√	√	√	√	
Abu Seman et al. (2019) [45]					√		√
Jun et al. (2019) [46]	√	√	√				√
Qin et al. (2022) [22]	√				√		√
Yang et al. (2022) [24]	√	√	√	√			√
This research	√	√	√	√	√	√	√

Note: Summary by authors according to [22,23,24,45,46].

**Table 2:** Target literature code table.

Author Year	Outcome	Sample Size	Fisher’s *Z*	Standard Error	Region
Abu Seman et al. (2019) [45]	Economic, Environmental Performance	123	0.427, 0.420	0.091	Malaysia
Akhtar et al. (2021) [74]	Economic	477	0.428	0.046	Pakistan
Frempong et al. (2021) [75]	Social	243	0.459	0.065	Ghana
Han et al. (2021) [76]	Technological, Economic	210	0.751, 0.490	0.070	China
Iqbal et al. (2021) [77]	Environmental Performance	245	0.375	0.064	Various
Jum’a et al. (2021) [78]	Social	392	0.642	0.051	Jordan
Jun et al. (2019) [46]	Political, Economic, Technological	288	0.968, 0.912, 0.354	0.059	Pakistan
Li et al. (2020) [60]	Environmental Performance	229	0.623	0.067	China
Roh et al.(2021) [57]	Political, Technological	1203	0.229, 0.282	0.029	South Korea
Scarpellini et al. (2018) [79]	Political	87	0.415	0.109	Spain
Segarra-Oña et al. (2014) [80]	Economic	223	0.589	0.067	Spain
Shahzad et al. (2021) [81]	Social	393	0.420	0.051	Pakistan
Singh et al. (2020) [30]	Environmental Performance	309	0.842	0.057	The United Arab Emirates
Sobaih et al. (2020) [82]	Environmental Performance	525	0.733	0.044	Egypt
Waqas et al. (2021) [83]	Social, Environmental Performance	294	0.209, 0.277	0.059	China
Zhou et al. (2020) [84]	Technological	230	0.706	0.066	China

**Table 3:** Bias test.

Outcome	Rosenthal‘S Fail-Safe N			Begg and Mazumdar Rank Correlation *p*-Value	Egger’s Regression (2-Tailed)		
	*Z*-Value	*p*-Value	α		*p*-Value	Low Limit	Upper Limit
Economic	22.316	<0.001	0.050	1.000	0.773	−25.687	31.333
Political	15.663	<0.001	0.050	0.602	0.583	−121.627	137.267
Social	16.390	<0.001	0.050	1.000	0.104	−76.040	54.621
Technological	16.717	<0.001	0.050	0.174	0.181	−7.469	20.661
Environmental Performance	22.388	<0.001	0.050	0.573	0.375	−30.598	15.437

**Table 4:** Total sample heterogeneity test.

Model	k	Combined Effect Size	95% Confidence Interval		Q-Value	df	*p*-Value	I^2^
			LL	UL				
Fixed	22	0.470	0.448	0.492	405.242	21	<0.001	94.818
Random	22	0.525	0.425	0.625				

Note: LL = 95% Confidence Interval Low Limit, UL = 95% Confidence Interval Upper Limit.

**Table 5:** The main effect results.

Category	Outcome	k	Combined Effect Size	95% CI		*p*-Value	Total Effect Size
				LL	UL		
Environment	Economic	5	0.635	0.418	0.852	<0.001	0.521
	Political	2	0.519	0.035	1.003	<0.001	
	Social	5	0.451	0.299	0.603	<0.001	
	Technological	4	0.453	0.268	0.638	<0.001	
Enterprise	Environmental Performance	6	0.536	0.334	0.739	<0.001	0.536

Note: LL = 95% Confidence Interval Low Limit, UL = 95% Confidence Interval Upper Limit.

**Table 6:** Moderating effect results.

Region	k	Effect Size	95% CI		2-Tailed Test: *p*-Value	Q Statistics			I^2^	τ^2^
			LL	UL		Q-Value	df	*p*-Value		
China	6	0.371	0.333	0.410	<0.001	73.592	5	<0.001	93.206	0.036
Egypt	1	0.733	0.647	0.819	<0.001	0.000	0	>0.050	0.000	0.000
Ghana	1	0.490	0.354	0.626	<0.001	0.000	0	>0.050	0.000	0.032
Jordan	1	0.912	0.796	1.028	<0.001	0.000	0	>0.050	0.000	0.000
Malaysia	2	0.439	0.365	0.512	<0.001	0.146	1	>0.050	0.000	0.000
Pakistan	5	0.362	0.321	0.403	<0.001	69.338	4	<0.001	94.231	0.042
South Korea	2	0.541	0.428	0.653	<0.001	1.828	1	>0.050	45.297	0.007
Spain	2	0.424	0.297	0.550	<0.001	0.003	1	>0.050	0.000	0.000
The United Arab Emirates	1	0.842	0.730	0.954	<0.001	0.000	0	>0.050	0.000	0.000
Various	1	0.968	0.852	1.084	<0.001	0.000	0	>0.050	0.000	0.000
Total within						144.907	12	<0.001		
Total between						260.334	9	<0.001		

Note: LL = 95% Confidence Interval Low Limit, UL = 95% Confidence Interval Upper Limit.

## Data Availability

Not applicable.
